# Research on Deep Learning-Based Method for Bearing Fault Diagnosis in TENG Under Wear Conditions

**DOI:** 10.3390/mi17080918

**Published:** 2026-07-30

**Authors:** Zhihang Li, Weili Tang, Qingshan Duan, Xinxin Li, Mingchun Wang

**Affiliations:** 1 School of Mechanical Engineering, Guangxi University, Nanning 530004, China; 2311301034@st.gxu.edu.cn (Z.L.); lixinx@gxu.edu.cn (X.L.); wangmc@st.gxu.edu.cn (M.W.); 2 School of Light Industry and Food Engineering, Guangxi University, Nanning 530004, China; qs_duan@gxu.edu.cn

**Keywords:** triboelectric nanogenerator, wear and degradation, fault diagnosis, deep learning, incremental learning

## Abstract

The Triboelectric Nanogenerator (TENG), as an emerging self-powered sensor, is widely used in the field of rotating machinery bearing fault diagnosis. Due to its working principle based on frictional electrification and electrostatic induction effects, the surface morphology and charge transfer efficiency of the friction layer have a significant impact on the output performance of TENGs. Under long-term mechanical motion, the friction layer may experience wear, and continuous wear can lead to surface morphology damage and even damage to the friction layer structure, gradually destroying the TENG’s signal acquisition and output capabilities, causing signal degradation and bearing fault feature deviation, which results in a decrease in bearing fault diagnosis accuracy. Traditional solutions focus on material properties and structure. This article derives the mechanism of the influence of friction layer thickness on the output signal through the TENG output voltage formula and simulates different degrees of wear with friction layers of different thicknesses to conduct deep learning-based bearing fault diagnosis experiments. The experimental results show that although the CNN model can recognize TENG signals well for bearing fault classification, the bearing fault features of the worn signals shift, and the accuracy of CNN diagnosis decreases. The introduction of a one-dimensional self-attention-enhanced convolutional neural network model and an incremental learning method improved the accuracy of bearing fault diagnosis after wear and tear. This study provides theoretical support and practical solutions for long-term, stable bearing fault diagnosis in TENG under wear conditions.

## 1. Introduction

The reliabloperation of rotating machinery is fundamental to modern industrial systems, where unexpected failures can lead to significant economic losses and safety hazards. In this context, condition monitoring and bearing fault diagnosis have become indispensable. Emerging as a self-powered sensing technology, the Triboelectric Nanogenerator (TENG) has garnered significant attention for bearing fault diagnosis due to its flexibility, simple structure, low cost, and robust environmental adaptability [1,2,3,4,5,6]. Unlike conventional sensors, TENGs harvest energy from their operating environment, theoretically enabling long-term, maintenance-free monitoring.

However, the operational principle of TENGs—relying on contact electrification and electrostatic induction—presents an inherent paradox. High frictional contact is necessary for charge generation, yet continuous mechanical motion inevitably causes wear on the friction layer. This wear degrades the surface morphology and charge transfer efficiency, leading to signal degradation and a subsequent decline in diagnostic accuracy [7]. Consequently, this can result in misdiagnosis of equipment health, threatening the safety and reliability of the entire mechanical system.

Addressing the trade-off between sustained contact for electrification and minimizing wear for longevity is a central challenge in the field. Current research has pursued three primary strategies. The first focuses on material reinforcement, where the friction layer is strengthened using composite materials or nanoparticles [8]. A second, evolving strategy aims to impart self-healing properties to the friction layer [9,10,11,12,13,14,15,16]. The third approach aims to directly enhance wear resistance, for example, [17,18,19], through the development of advanced composite films such as PVC/MoS [20] or by incorporating diamond-like coatings to reduce the friction coefficient [21,22]. A second major strategy involves the use of lubricating greases and additives. While lubrication reduces friction and wear, some studies have surprisingly shown that certain lubricants can simultaneously improve the electrification performance of TENGs [23,24,25,26,27]. For example, squalene has been identified as an additive that enhances both wear resistance and triboelectric output under light loads [28,29]. A third strategy involves the structural optimization of the TENG device itself to balance charge generation and wear reduction [30,31,32,33,34,35].

Despite these advances, each solution faces practical limitations. Material and structural optimizations often involve high preparation costs, complex design processes, and long development times, with solutions rarely being transferable across different application scenarios. This significantly hinders the widespread industrial deployment of TENG-based diagnostic systems.

In response to these challenges, this work presents a fundamentally different approach that integrates materials science with data-driven deep learning. The main aim is to mitigate the effects of TENG wear without requiring complex material or structural changes. Specifically, we derive the theoretical relationship governing how TENG output voltage is affected by friction layer thickness—using thickness reduction as a proxy for mechanical wear. We then systematically collect bearing fault-related data under simulated wear conditions. The principal conclusions are twofold: First, we demonstrate that incremental learning can effectively compensate for the decline in diagnostic accuracy caused by progressive wear. Second, we propose a one-dimensional self-attention-enhanced convolutional neural network (1D Self-Attention CNN) as a benchmark model, which further improves diagnostic reliability. Together, these findings provide a theoretical and practical pathway for stable, long-term bearing fault diagnosis using TENG sensors under complex and realistic wear conditions.

## 2. Related Theoretical Foundations

### 2.1. Working Mechanism and Structure

**Figure 1:**
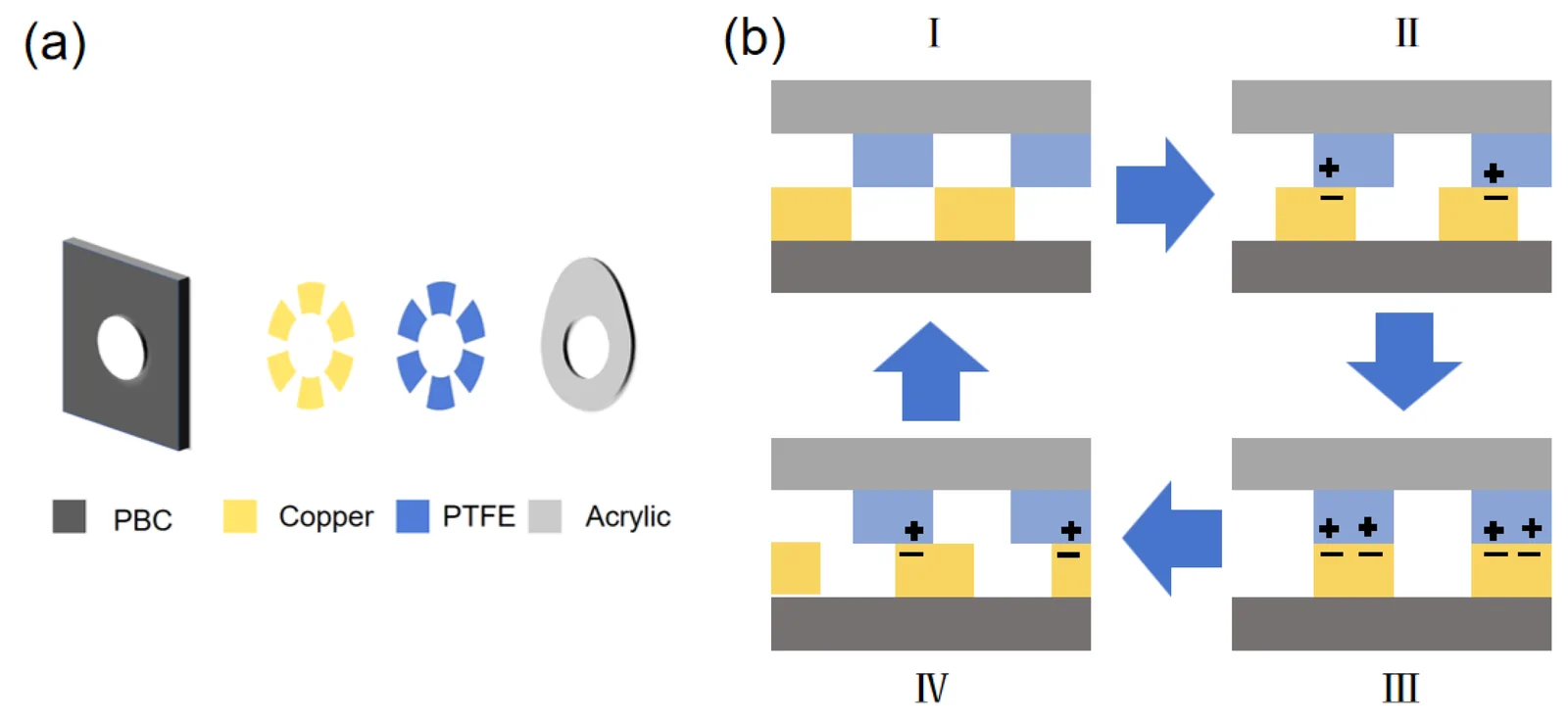
TENG structure and working principle diagram. (**a**) Structural schematic diagram, (**b**) working principle diagram, I–IV represent the four stages in the TENG working process, “+” and “−” represent the surface charge properties carried by TENG, and different colors represent different materials, which are the same as in Figure a.

The working principle of the triboelectric nanogenerator (TENG) is based on frictional electrification and electrostatic induction effects. The structural parameters of the TENG friction layer are a center hole radius of 12.75 mm, an outer ring radius of 33 mm, a thickness of 2 mm, a cam structure radial length of 45 mm, and an angle of 120°. The overall size of the electrode layer is 50 mm × 50 mm, with a center hole radius of 12.75 mm and a thickness of 2 mm. PTFE and copper foil are alternately arranged in 12 equal circles, and 6 pieces are selected and pasted on the surfaces of the friction layer and electrode layer, respectively. The structure and working principle of the TENG are shown in Figure 1a. The TENG structure consists of copper electrodes on the PCB board, PTFE film, and acrylic board. The working principle is shown in Figure 1b. In the first stage, the upper and lower electrodes are separated and do not generate relative charges. In the second stage, they come into contact with each other and generate an opposite amount of charges, resulting in induced currents in the external circuit. In the third stage, complete contact generates an opposite amount of charge, but the charge reaches equilibrium and no induced current is generated. In the fourth stage, the upper and lower electrodes begin to separate, and the change in charge generates induced current in the external circuit, finally returning to the first stage to complete the power generation cycle.

The independent layer TENG used in this article adopts a single-electrode working mode, where one electrode remains stationary and the other friction layer undergoes periodic contact and separation with mechanical rotation, ignoring edge effects and assuming uniform charge distribution. Its working principle can be equivalent to a parallel plate capacitor, and its theoretical formula is as follows:

(1) $$V=\frac{Q}{C}=\frac{\sigma S}{C},$$

(2) $$C=\frac{\varepsilon S}{d},$$

Here, *S* is the contact area, *Q* is the surface charge, *C* is the capacitance, σ is the surface charge density, *V* is the open circuit voltage, and *d* is the thickness of the friction layer. Substitute the formula into:

(3) $$V=\frac{\sigma d}{\varepsilon }.$$

From the above formula, it can be concluded that the open circuit voltage *V* of the TENG is approximately linearly positively correlated with the thickness *d* of the friction layer. During the wear process, the thickness of the friction layer changes, and the thickness *d* continues to decrease, resulting in a regular decrease in the output voltage of the frictional electrical signal. The thickness *d* is the main influencing factor of wear, while its surface morphology changes due to wear, leading to changes in the surface charge density σ of the TENG. However, due to its small order of magnitude, it is ignored here.

### 2.2. Incremental Learning

**Figure 2:**
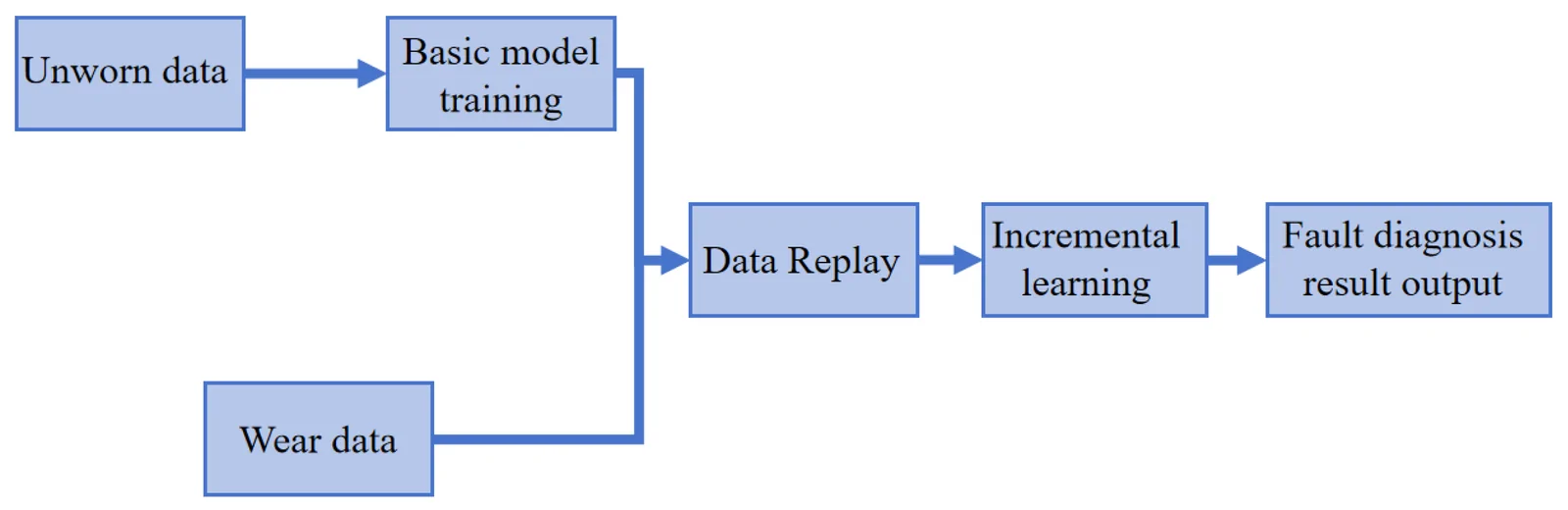
Schematic diagram of incremental learning with data replay.

Due to the interference of new data on already learned knowledge during the generation process, it can lead to catastrophic forgetting. In recent years, scholars at home and abroad have proposed various methods to alleviate catastrophic forgetting and improve the performance of incremental learning. According to the main mechanisms for alleviating catastrophic forgetting, they can be divided into the following: incremental learning methods based on parameter regularization, incremental learning methods based on data replay, incremental learning methods based on knowledge distillation, and incremental learning methods based on dynamic extension network architecture. The incremental learning method used in this article falls under incremental learning strategy based on data replay. The schematic diagram is shown in Figure 2:

When monitoring the operating conditions of rotating machinery related to long-term TENG operation, TENG wear leads to a continuous degradation of signals and a decrease in bearing fault accuracy. While learning the characteristics of wear conditions through new wear data, the model is prone to forgetting the bearing fault knowledge learned under unworn conditions, resulting in catastrophic forgetting problems. As such, we introduced representative old working condition samples and used a mixed training method of new and old data during the fine-tuning stage, allowing the model to learn new wear features while revisiting existing bearing fault knowledge and maintaining its ability to recognize existing bearing fault features. At the same time, with the combination of extremely low learning rates and hierarchical fine-tuning strategies, the model parameters were updated while maximizing the retention of learned shared features, achieving dynamic balance and stable updates of new and old knowledge, thereby effectively improving the model’s continuous diagnostic performance under long-term monitoring and changes in wear and tear levels.

## 3. Experiments and Analysis

### 3.1. Experimental System Construction

**Figure 3:**
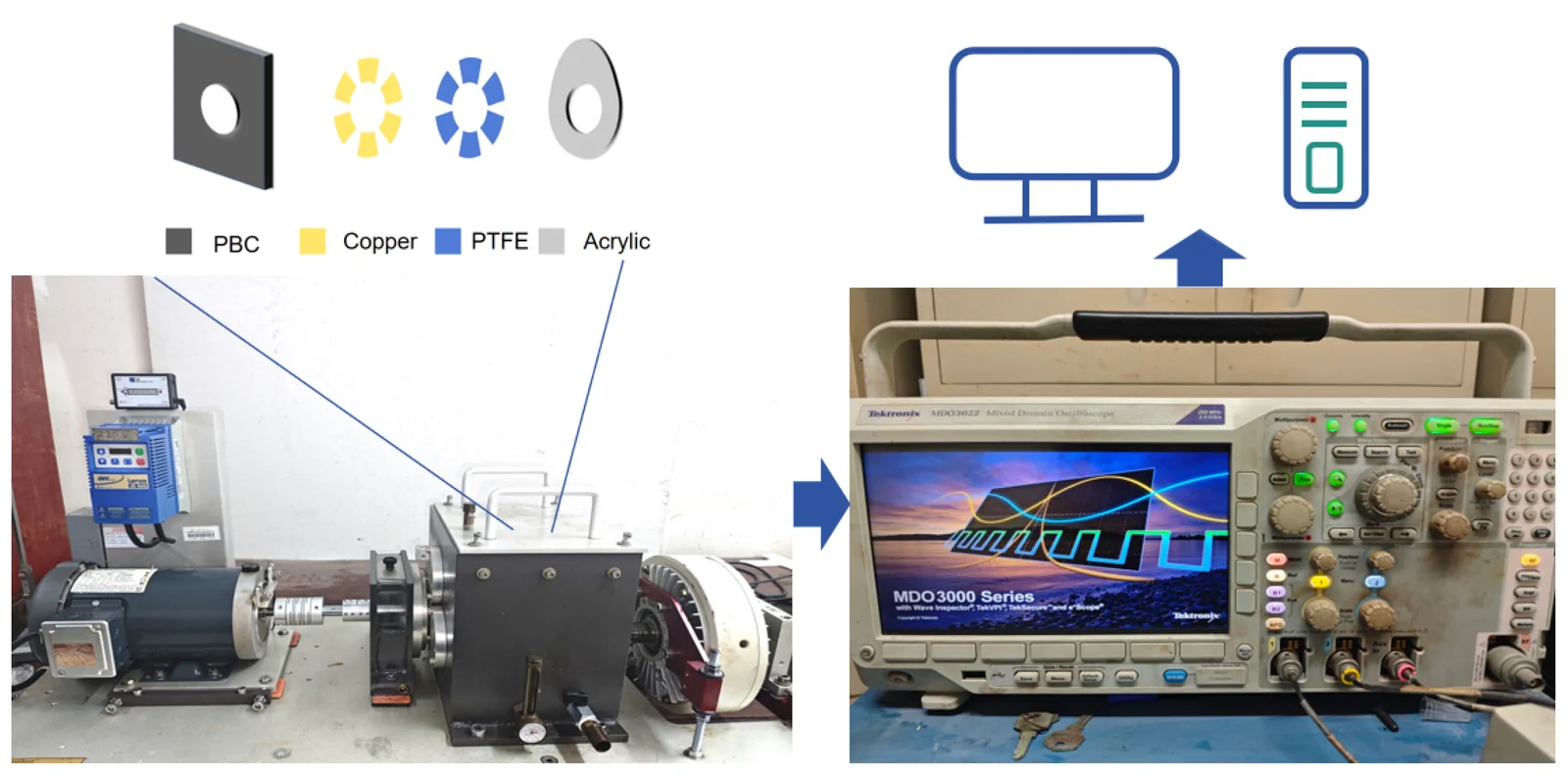
Experimental setup and flowchart: TENG test rig, oscilloscope, and signal processing PC.

The experimental system includes the following four modules: DDS rotating machinery bearing fault diagnosis test bench TENG, signal acquisition device oscilloscope and signal processing PC. The experimental flowchart is shown in Figure 3. The motor of the DDS rotating machinery bearing fault test bench drives the gearbox to operate, and the TENG friction layer rotates accordingly, generating relative motion and frictional charges. The oscilloscope connected to the TENG collects relevant signals, which finally processes and analyzes the signals through software on the computer.

### 3.2. Experimental Plan Design

This article uses friction layers of different thicknesses to simulate the different degrees of wear of TENGs: the unworn state corresponds to an original friction layer thickness of 1 mm; slight wear corresponds to a thickness of 0.8 mm; and severe wear corresponds to a thickness of 0.5 mm. By controlling the thickness of the friction layer, the output signals of TENGs at different degrees of wear during operation are simulated. Combined with relevant characteristic indicators, the degradation law of signal characteristics under different degrees of wear is analyzed, laying a theoretical foundation for subsequent bearing fault diagnosis experiments. Table 1 outlines the design of the multi-condition experimental scheme for TENG wear.

**Table 1:** TENG wear multi-condition experimental plan design.

Number	Thickness	Wear Degree	Bearing Fault Type	Speed (rpm)	Number of Categories
1	0.5 mm	Severely worn	Inner ring fault, outer ring fault, ball fault, health	300, 600, 900	12
2	0.8 mm	Mild wear	Inner ring fault, outer ring fault, ball fault, health	300, 600, 900	12
3	1 mm	Unworn	Inner ring fault, outer ring fault, ball fault, health	300, 600, 900	12
Total					36

**Figure 4:**
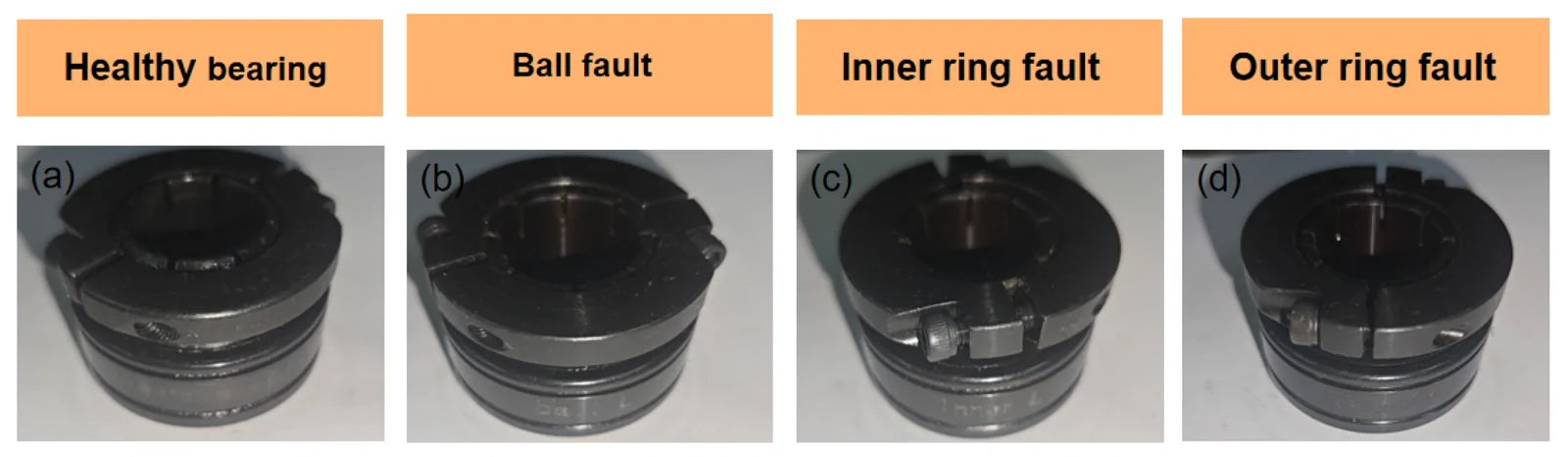
Bearing display diagram: (**a**) Healthy bearing; (**b**) ball fault bearing; (**c**) inner ring fault bearing; (**d**) outer ring fault bearing.

This article uses different types of prefabricated faulty bearings, as shown in Figure 4. The four types of bearings used in the experiment include healthy, ball failure, inner ring failure, and outer ring failure.

### 3.3. Data Collection and Analysis

**Figure 5:**
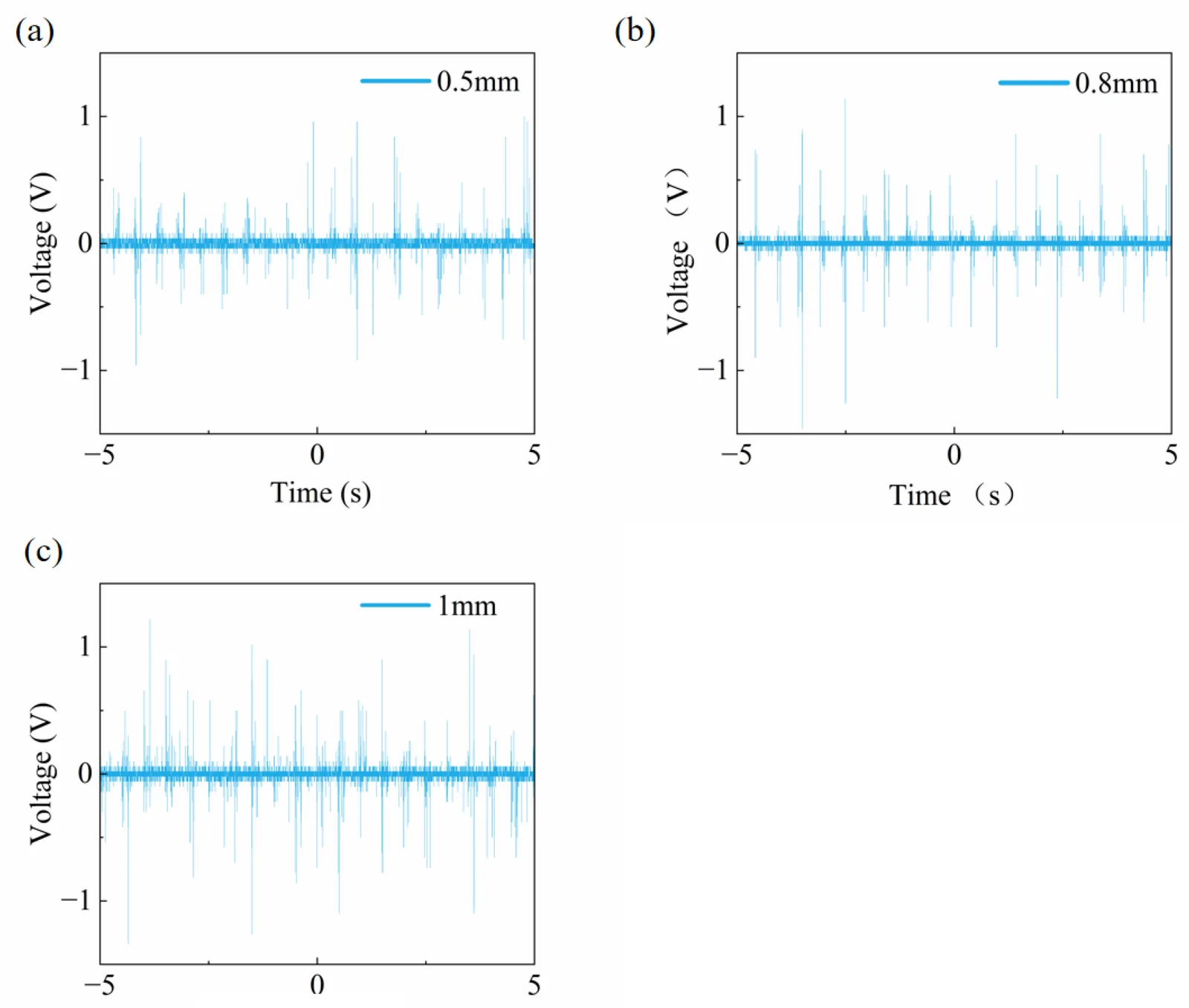
Voltage signal curve of TENG under different wear thicknesses: (**a**) 0.5 mm; (**b**) 0.8 mm; (**c**) 1 mm.

According to the experimental plan, TENG output voltage signals were collected under different operating conditions, including 36 types of signals for 4 types of bearings, 3 speeds, and 3 degrees of wear. Using a gear fault diagnosis test bench as the operating system of the bearing, a frictional piezoelectric sensor as the signal generation device, and an oscilloscope as the acquisition device, relevant voltage signals were collected with a sampling frequency set to 1 kHz for a total of 10 s. The collected TENG voltage signals of different thicknesses at 900 rpm are shown in Figure 5. The signals change over time and exhibit significant fluctuations with periodic patterns, which are consistent with the working principle of TENGs. Comparing the voltage signals of TENGs with different thicknesses, it was found that the peak changes in voltage signals across the different wear thicknesses were difficult to visually observe. This may be due to the small contact area S of the TENG used, and the voltage peak changes caused by the thickness variation of 0.2 mm–0.5 mm are difficult to visually represent, especially on the curve graph. At the same time, due to the difficulty in controlling the period of the relevant signals during acquisition, their signal spectra are difficult to correspond one-to-one with their working states, and it is even more difficult to compare and analyze them with other signals in different working states.

**Figure 6:**
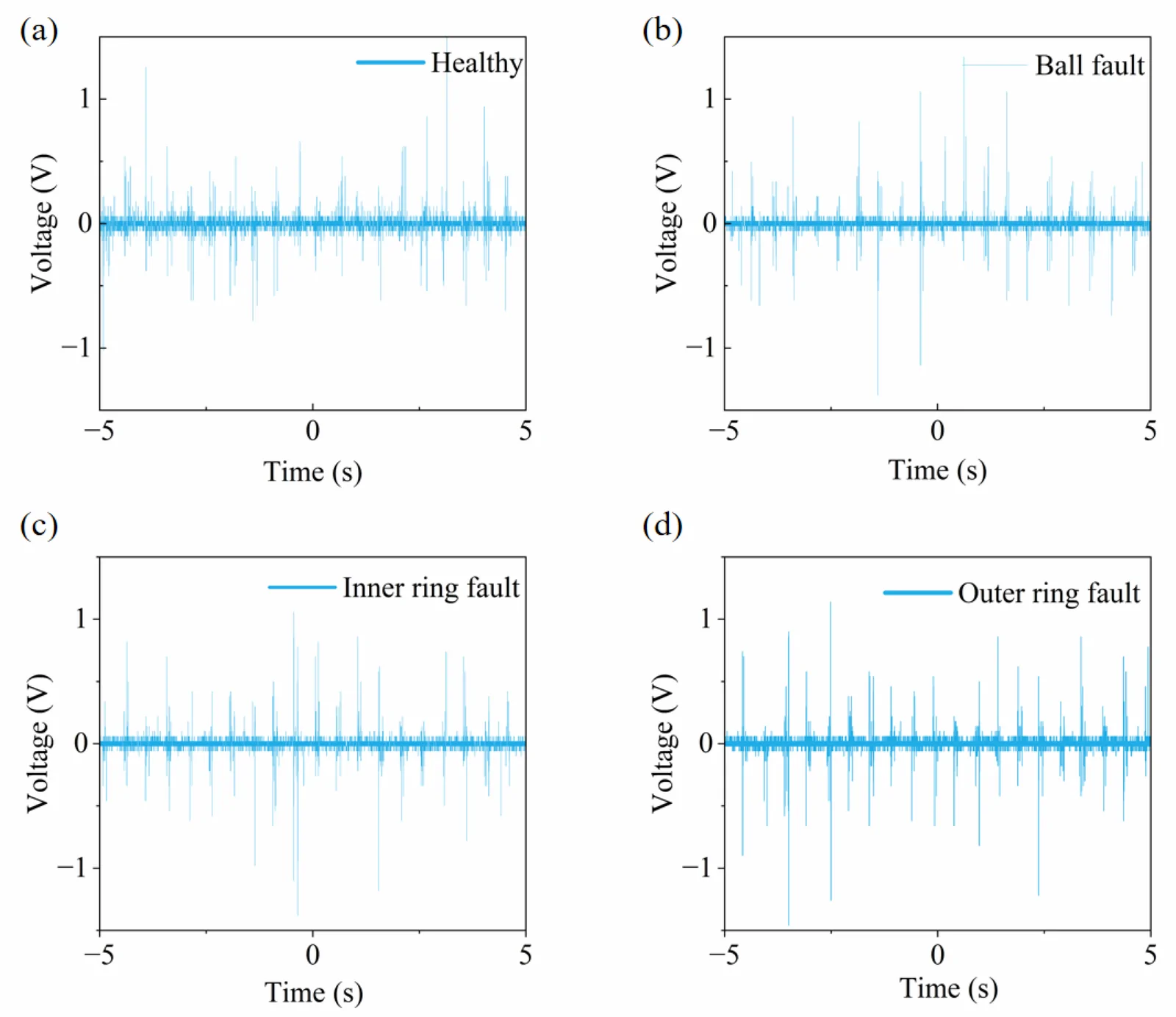
Voltage signal curves of TENG under different bearing fault types: (**a**) Healthy; (**b**) ball fault; (**c**) inner ring fault; (**d**) outer ring fault.

Therefore, it was necessary to use other relevant signal indicators to more intuitively represent the changes in TENG voltage signals in relation to the degree of wear. Figure 6a–d show the TENG voltage signals generated by different types of faulty bearings.As TENG voltage signals are mainly one-dimensional voltage signals, they exhibit a time–voltage curve. Therefore, selecting appropriate time-domain signal characteristic indicators for quantitative analysis can effectively reveal the evolution law of TENG voltage signals alongside the degree of component wear and provide theoretical basis and data support for signal characteristic analysis under different wear states.

The macroscopic variation pattern of the original voltage waveform was not obvious, and it was difficult to accurately identify the degree of wear based on intuitive curve characteristics. Further analysis was conducted on the time-domain signal characteristics, such as the average absolute value, standard deviation, peak value, and root mean square of the signal. The relevant results are shown in Table 2. As the wear thickness gradually increases from 0.5 mm to 1 mm, the characteristics show an overall upward trend. Although wear did not significantly change the macroscopic characteristics of the signal, it significantly altered the intrinsic information of the signal. The characteristic values of its signal underwent significant changes, and showed regular variations alongside thickness changes. The mean absolute value of 0.0368 at 1 mm was only 0.0271 at 0.5 mm, indicating that simulating the degree of wear alongside thickness changes was feasible. As the degree of wear increased, the overall amplitude level of the signal decreased, and the TENG had a sensitive response to changes in the state of the contact surface. The changes in both standard deviation and root mean square demonstrate that the energy of the signal decreases with the degree of wear. The decrease in peak value also indicates that the impulse component of the relevant signal decreased. We verified the feasibility of simulating TENG wear-induced signal degradation through thickness simulation, providing a research basis for further bearing fault diagnosis experiments in the future. A limitation of this study is that it did not conduct microscopic morphology characterization and complete electrical parameter testing on the friction layer. Subsequent work will combine SEM, surface roughness testing, and complete electrical parameter measurement to further explore the evolution of TENG wear morphology and its impact mechanism on output signals.

## 4. Experimental Results and Analysis of Bearing Fault Diagnosis

### 4.1. Experimental Environment and Parameter Settings

Using version Python 3.8.19, the model was trained on a Windows 11 system with a GeForce GTX 4070 GPU. The sampling dataset contained 1000 data points per file, totaling 36 different operating condition files. Each sample contained 800 data points, with a sampling interval set to 100 to ensure data independence and validity. The dataset classification targeted 4 categories—healthy, ball faults, inner ring faults, and outer ring faults—with a total of 93 samples per category and 372 samples in total. During training, the training dataset and testing dataset were divided in a ratio of 8:2. The training parameters related to the model were set as follows: Loss function—The CrossEntropy Loss function, as CrossEntropy Loss is suitable for multi-class bearing fault diagnosis tasks; Optimizer—The Adam optimizer adopted a layered learning rate strategy during incremental learning training to improve the model’s convergence speed and generalization ability; Evaluation indicators—Accuracy, Precision, Recall, and F1 score, which were used for the comprehensive evaluation of model diagnostic performance. All together, this study proposes a novel TENG sensor structure. The various parameters during the training process are shown in Table 3.

**Figure 7:**
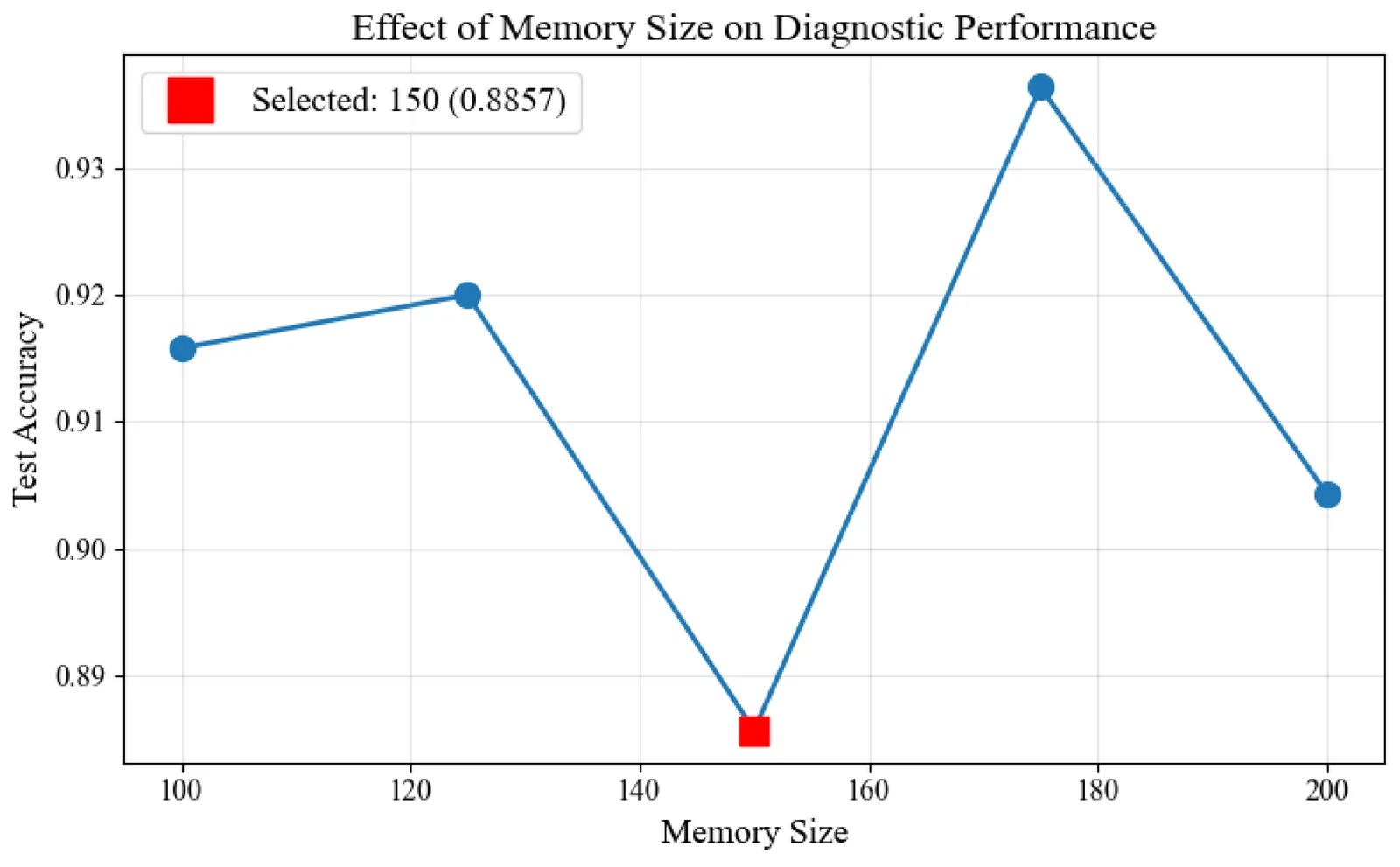
Accuracy comparison under different memory sizes (100, 125, 150, 175, and 200) for incremental learning.

From Figure 7, it can be seen that a memory set size of 150 exhibits the worst performance, as it resides in the critical zone where the conflict between new and old knowledge is most intense, and it represents the memory level with the highest risk of catastrophic forgetting. A size that is too small results in insufficient historical information, while one too large introduces redundant noise. If incremental learning methods can still achieve acceptable performance at the most challenging point of 150, then they naturally possess more robust performance guarantees at other memory scales. Selecting this point as the standard configuration is equivalent to validating the method’s robustness under the most stringent conditions.

### 4.2. Results and Analysis

Bearing fault diagnosis and classification experiments were conducted using unworn data combined with the CNN and 1D Self-Attention CNN models. In order to better quantify the performance of the model, accuracy, precision, recall, and F1-measure were used as performance evaluation indicators. The experimental results are shown in Table 4.

The trained CNN model was directly tested using TENG data under wear conditions without any optimization methods. The accuracy of bearing fault diagnosis was only about 19%, indicating that the signal degradation caused by TENG wear seriously affected the recognition of the model, and the model could not accurately identify the bearing fault characteristics in the wear signal.

The trained 1D Self-Attention CNN model was directly tested using TENG data under wear conditions without any optimization strategy. The diagnostic accuracy was only about 56%. Although wear degradation affected the diagnostic ability of the model, the replaced baseline model was better able to identify the same bearing fault feature information contained in the relevant signals compared to a simple CNN model and had stronger extraction ability. Table 5 shows that the signal degradation caused by TENG friction-layer wear has a significant impact on the accuracy of bearing fault diagnosis.

**Figure 8:**
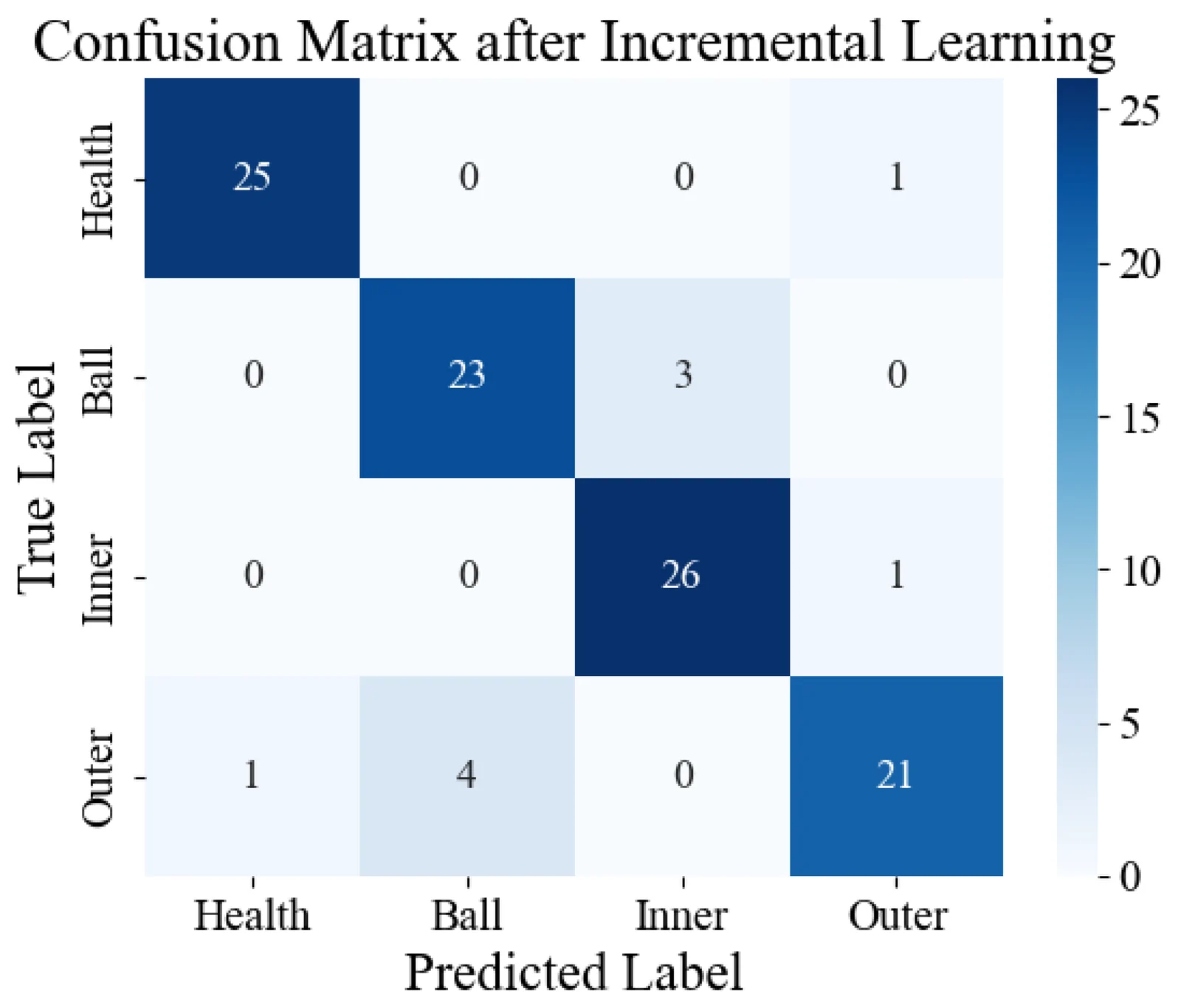
Confusion matrix diagram for incremental learning.

It can be seen that the TENG wear leads to the attenuation of frictional electrical signals and feature shift, resulting in a rapid decrease in the accuracy of TENG bearing fault diagnosis. The CNN model had an accuracy of only 29%, a recall rate of 28.75%, and an F1 score of 26.25%, basically losing its effective diagnostic ability. Although the accuracy and metrics of the 1D Self-Attention CNN model declined, it still outperformed the CNN model, verifying the adaptability of the 1D Self-Attention CNN model used. This may be due to the attention mechanism effectively alleviating signal degradation through global connections, capturing and identifying residual bearing fault information. The CNN model was optimized using incremental learning methods, fine-tuned with minimal learning rates, and trained with a mixture of new and old data to ensure that it could adapt to worn-bearing fault features without losing unworn feature information, effectively alleviating the accuracy decline caused by TENG wear. From Table 5, Table 6 and Table 7, it can be seen that the accuracy increased from 19% to 45.75%. The incremental learning method was proven to effectively alleviate the accuracy decline caused by wear, which may be due to the limited feature extraction ability of CNN models and the lack of global correlation ability, making it difficult to capture the small bearing fault features of TENG wear signals after degradation. The 1D Self-Attention CNN model was optimized and trained using incremental learning methods, with minimal learning-rate fine-tuning and mixed training of new and old data to ensure that it could adapt to worn bearing fault features without losing unworn feature information, effectively alleviating the accuracy decline caused by TENG wear. From Table 5, Table 6 and Table 7, it can be seen that the accuracy increased from 59% to 86.66%. The incremental learning method effectively alleviated the problem of accuracy decline caused by wear and tear. The confusion matrix for incremental learning is shown in Figure 8. The precise identification of healthy samples indicated that the model could distinguish the characteristics between normal signals and bearing fault signals, effectively identifying bearing faults, but not confuse bearing faults. There were 1–2 recognition errors in the ball faults and inner ring faults categories, which could determine the characterization of ball faults and inner ring faults. However, the recognition effectiveness for outer ring faults was not good, resulting in multiple misjudgments. This suggests that outer ring faults are easily confused with ball faults and inner ring faults. In terms of bearing mechanism, the impact of outer ring faults is transmitted through the rolling elements and coupled with the vibration components of the rolling elements and inner ring. The disturbance of working conditions further causes the mixing of fault characteristics. At the TENG-sensing level, the distance between the outer ring and the sensor resulted in significant attenuation of impact energy, making it difficult for the model to extract relevant fault features and leaving it prone to misjudgment.

In order to compare the effectiveness of incremental learning between the two models more clearly, the relevant data indicators are shown in Table 6.

Through the incremental learning method, both models improved the diagnostic accuracy after wear, which verified the effectiveness of the incremental learning method. At the same time, the comparison between the two models also showed that the 1D Self-Attention CNN model used is more suitable for the relevant incremental learning method and can achieve better results. This may be due to the self-attention mechanism’s ability to better capture weak bearing fault features, preserve global feature correlations, and still have stronger feature reuse and adaptive learning capabilities when the feature distribution deviates. Therefore, it performs better in fine-tuning and incremental updating processes. To clearly compare the diagnostic performance of the different models and training strategies, a comparison of experimental results is presented in Table 7.

From the comparison of accuracy of different models and training strategies in Table 7, it can be seen that the 1D Self-Attention CNN model used has better extraction ability compared to the CNN model, and has better accuracy under the same conditions, verifying that the self-attention mechanism can effectively extract key fault features to improve the accuracy of bearing fault diagnosis. At the same time, the accuracy improvement of 1D Self-Attention CNN compared to CNN is greater after wear, indicating that the attention mechanism can better adapt to incremental learning methods and is more suitable for bearing fault diagnosis tasks in TENG-sensor wear scenarios.

From Table 8, it can be seen that the standard 1D Self-Attention CNN achieves a recognition accuracy of 100% for wear data at both 600 rpm and 900 rpm, with a high accuracy of 98.67% at 300 rpm, indicating that the model itself has strong feature-extraction ability and generalization performance, and can adapt well to different speed conditions. After introducing incremental learning methods under wear conditions, the accuracy decreased slightly, with a smaller decrease at 900 rpm, possibly due to the small difference in the distribution of fault feature information between new and old data at high speeds.

### 4.3. Feature Visualization and Analysis

**Figure 9:**
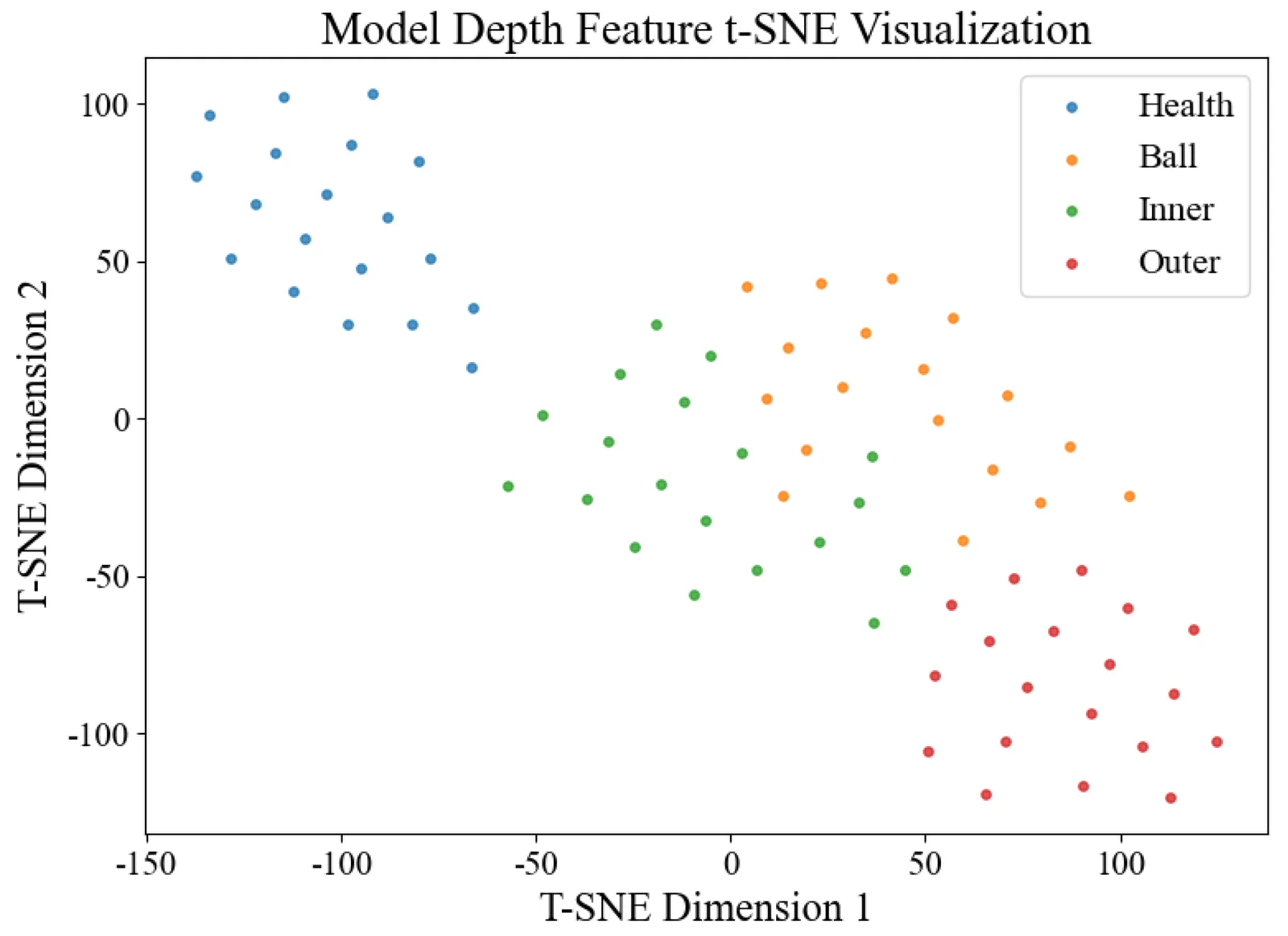
Data visualization of features for four unworn bearing conditions: Healthy, ball fault, inner ring fault, and outer ring fault.

The t-SNE feature visualization of the test set with unworn data is shown in Figure 9:

In response to the degradation of TENG signals and the decrease in bearing fault diagnosis accuracy caused by TENG wear, feature visualization of different wear stages can reveal the feature-stacking phenomenon caused by wear, the alignment and optimization effect of incremental learning features used, and the clustering and classification ability of features, and verify the effectiveness of the proposed model. In the absence of wear and tear, the pre-training effect of the model was very good, and the four types of bearing faults had clear boundaries and were fully recognized.

**Figure 10:**
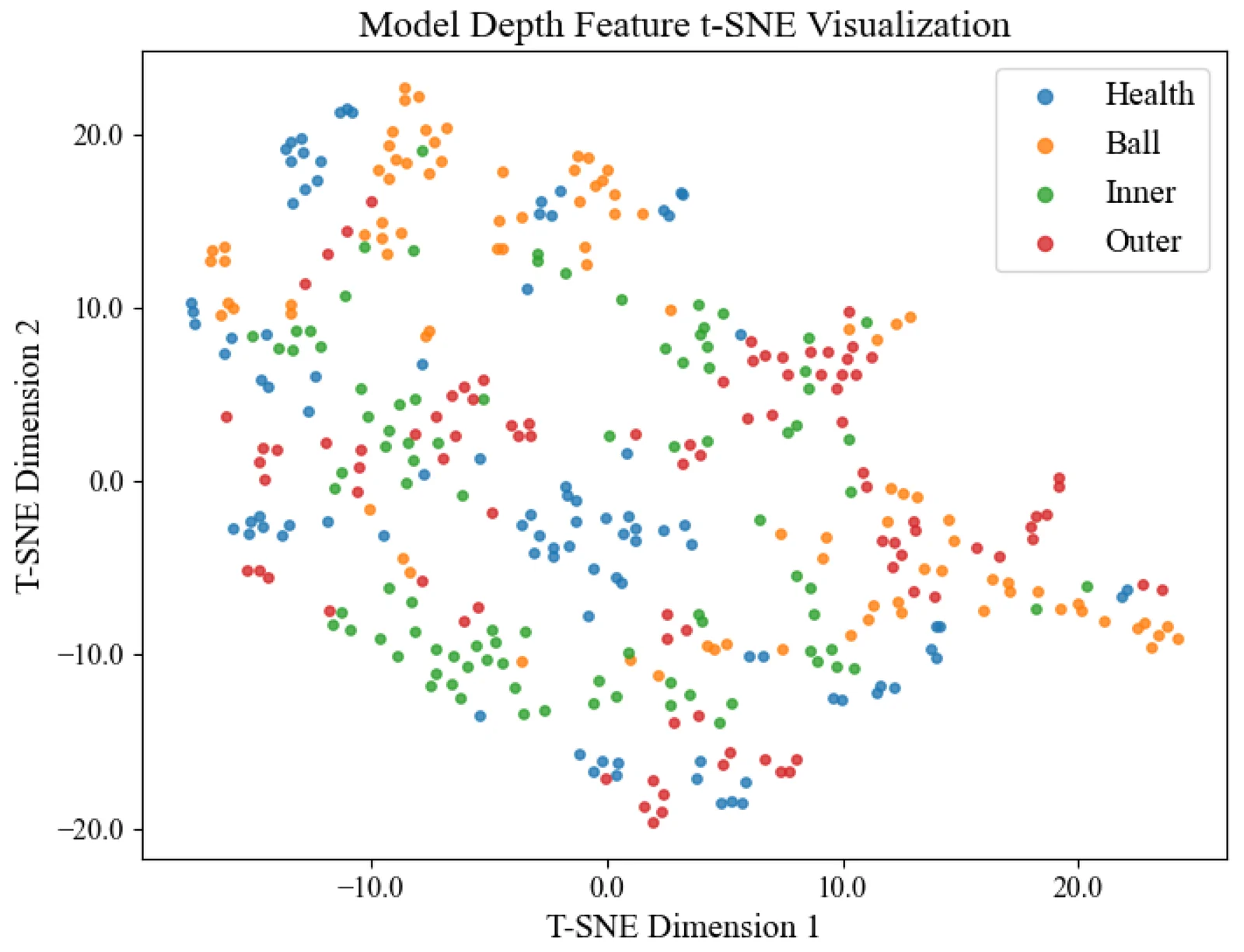
Feature visualization of migrating wear data to the model for four bearing conditions: Healthy, ball fault, inner ring fault, and outer ring fault.

The t-SNE feature visualization of the training results of the wear data model is shown in Figure 10. Due to the complete use of wear data and the large number of features, the healthy features in the figure are clustered into two main centers, leaving only scattered classification. This is because the distribution of wear data features is different from that of unworn data features, resulting in confusion and stacking of features, which is also the main reason for the decrease in classification accuracy. At the same time, the multi-cluster distribution of inner ring faults indicates that wear may have a significant impact on it. Ball faults and outer ring faults intersect extensively, making it difficult to form boundaries, which is reflected in the model’s misjudgment of such faults in the confusion matrix.

The feature-aliasing phenomenon generated in this figure verifies the domain shift caused by different features of the TENG wear signal, resulting in changes in signal amplitude, waveform, and other information, which leads to the destruction of the original fault distribution boundary. The originally highly discriminative fault features become similar after feature shift, making it difficult to distinguish from one another. This is an inherent problem in the research related to wear degradation and a deep-seated reason for the decrease in the accuracy of model fault diagnosis.

The healthy features are clustered in the lower right and left corners, with clear boundaries from the other three categories. The green distribution of faults in the inner circle is clear in the upper left area, and there is some overlap between the rolling element fault features and the outer circle fault features. The features are not completely separated, and the core area clusters well, but there are overlapping and stacking phenomena in the periphery. Overall, clear partitions are formed, and feature clustering is more pronounced compared to when incremental learning methods are not used.

**Figure 11:**
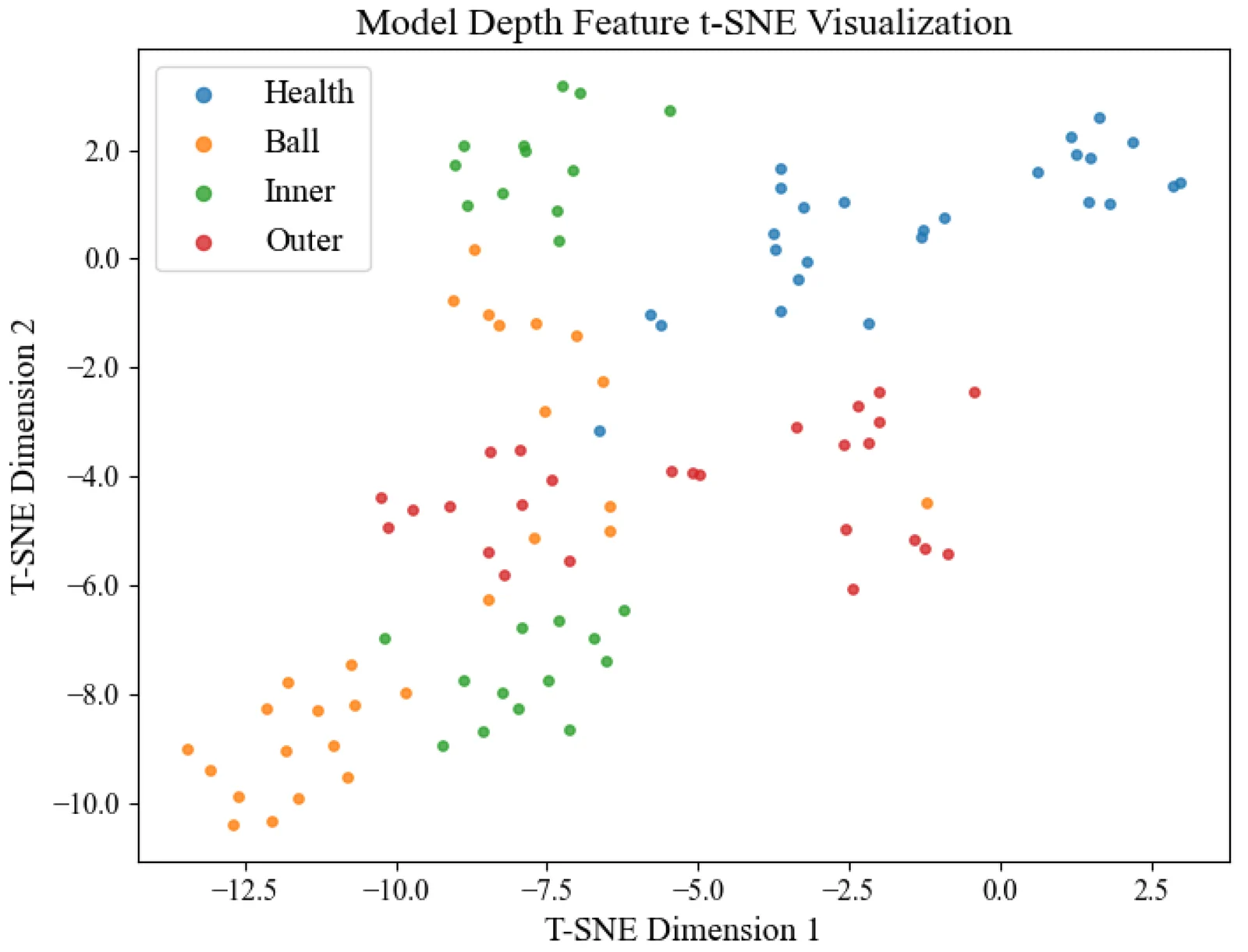
Feature visualization optimized through transfer and incremental learning for four bearing conditions: Healthy, ball fault, inner ring fault, and outer ring fault.

The strategy of incremental learning was verified to solve the problem of feature domain shift caused by wear. The originally separable fault features were mixed in high-dimensional space, which is the fundamental reason for the sharp drop in accuracy of the previous model on wear data. The results of incremental learning in Figure 11 show that the features of different bearing fault categories were pulled back to their respective clustering centers, achieving feature alignment and clustering. While retaining the recognition ability of old working conditions, it quickly adapted to the distribution characteristics of new signals of wear, providing feature-level support for TENG and other related sensors in long-term stable monitoring applications.

**Figure 12:**
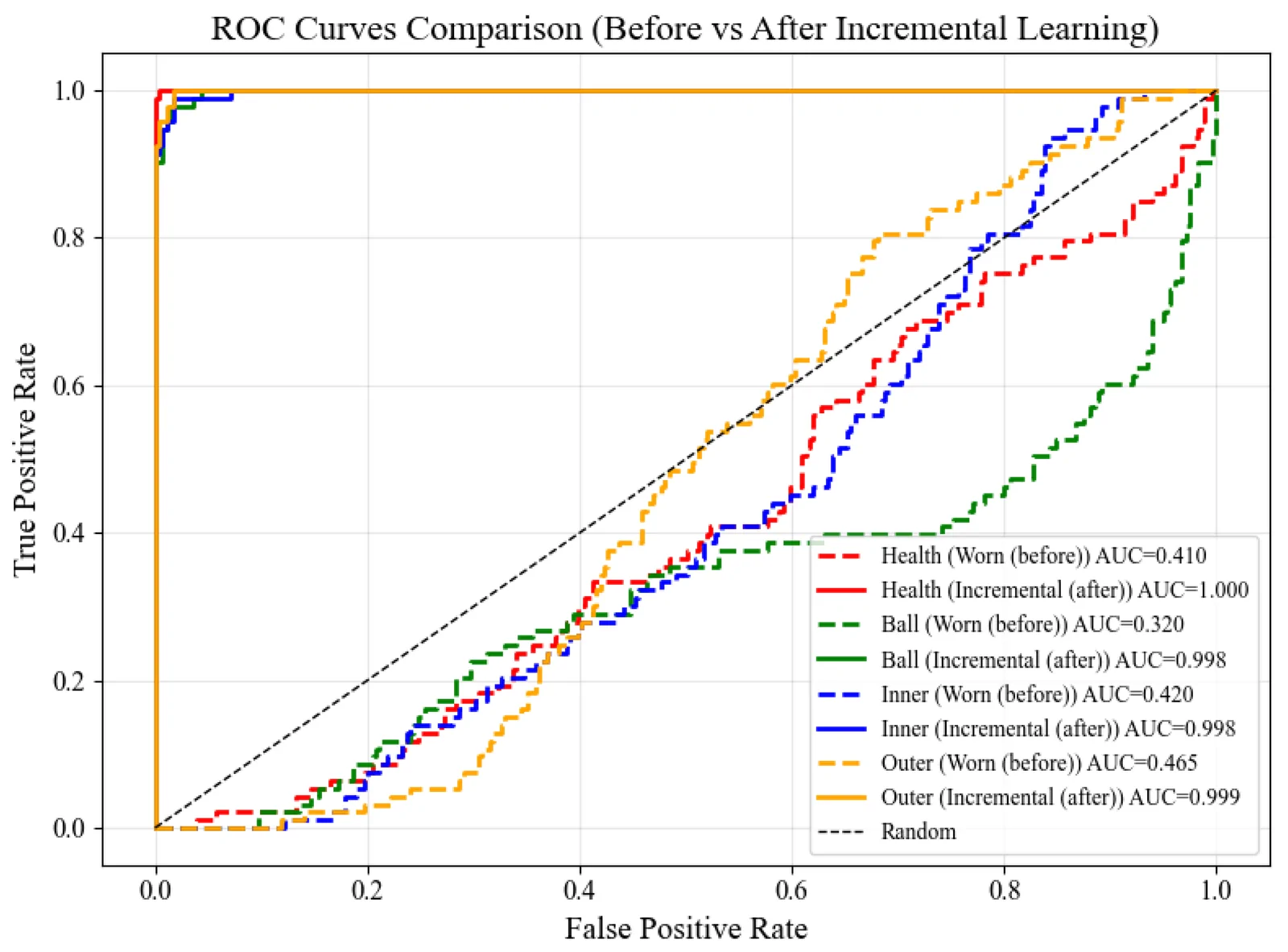
ROC curves for wear data from 1D Self-Attention CNN and its incremental learning variant.

The effect of combining 1D Self-Attention CNN with wear data is shown by the dashed line in Figure 12, indicating a significant decrease in overall performance. The healthy category is slightly higher than the diagonal, but the performance is far lower than that of the data without wear. The AUC values of the other three types of bearing faults are all below 0.5, indicating that the model has almost lost its ability to distinguish bearing faults on degraded signals. The diagnostic accuracy of the model is lower than random guessing, resulting in catastrophic forgetting and significant consequences. This indicates that the chaotic feature boundaries caused by signal wear destroyed the relevant bearing fault feature patterns learned in the pre-training stage, leading to a rapid decline in generalization ability and triggering catastrophic forgetting. It also highlights the necessity of conducting relevant incremental learning research. After introducing a hybrid incremental learning method based on data replay, further analysis was conducted, with the ROC curve and AUC value of the model are shown by the solid line in Figure 12. After incremental learning fine-tuning, the diagnostic performance of the model on wear data has significantly improved. The AUC values of the four types of bearing faults are all between 0.977 and 1, which are greatly improved. The model effect rebounded to the level before wear. At the same time, all four ROC curves are in line with the upper-left corner from the beginning, indicating that the model has almost no misjudgment, verifying that the proposed incremental learning strategy can effectively alleviate TENG wear. The problems of signal domain shift and feature overlap caused by friction and wear, as well as the data replay method, enable the model to quickly adapt to new wear signal distributions while retaining the recognition ability of old working conditions. The proposed method still has effectiveness and robustness under engineering wear conditions. It also provides a low-cost and replicable solution for the long-term stable monitoring needs of TENGs and other related sensors. Based on the above ROC curve and AUC values, it can be observed that the wear signal has chaotic feature boundaries caused by wear, which destroys the relevant bearing fault feature patterns learned in the pre-training stage, leading to a rapid decline in generalization ability and catastrophic forgetting. The proposed incremental learning strategy can effectively alleviate the problem of signal domain offset feature overlap caused by TENG friction wear, achieve wear adaptation, and improve diagnostic accuracy.

## 5. Conclusions

TENGs and other related sensors require frictional electrification to achieve signal transmission; as such, reducing friction is necessary to meet long-term monitoring needs. The existing solutions are costly, starting from optimizing material wear resistance, lubrication, and structural optimization. This article proposes a method that combines a self-attention mechanism and incremental learning. Wear simulation experiments and bearing fault diagnosis experiments showed that the proposed method can effectively alleviate the signal degradation and bearing fault feature shift caused by TENG wear, allowing the model to adapt to the worn features while not forgetting the original bearing fault features. Furthermore, the model improved the TENG sensor bearing fault diagnosis system, providing technical support for the long-term monitoring application of TENGs. 

## Figures and Tables

**Table 2:** Signal characteristic values at different thicknesses.

Thickness/Type	Average Absolute Value	Standard Deviation	Peak Value	Root Mean Square
0.5 mm	0.0271	0.0606	1.0	0.0606
0.8 mm	0.0325	0.066	1.27	0.0660
1 mm	0.0368	0.0762	1.34	0.0763

**Table 3:** Model training parameter settings.

Parameter Name	Parameter Value
Batch size	32
Initial learning rate	0.001
Extremely low learning rate	1 × 10^−5^
Random inactivation rate	0.7
Pre-training iterations	500
Iteration times	1000
Early stop basic model	200
Early stop iteration times	120
Sample size	500
Memory set	150
Hierarchical learning rate convolutional feature layers	1 × 10^−5^
Hierarchical learning rate fully connected classification layers	1 × 10^−4^

**Table 4:** Comparison of experimental results from different models.

Model	Precision (%)	Recall (%)	F1-Measure (%)
CNN	76.00	78.50	77.25
1D Self-Attention CNN	98.67	96.80	97.20

**Table 5:** Comparison of experimental results of different models under wear data.

Model	Precision (%)	Recall (%)	F1-Measure (%)
CNN	29	28.75	26.25
1D Self-Attention CNN	56	46	40

**Table 6:** Comparison of experimental results of different models under incremental learning methods.

Model	Precision (%)	Recall (%)	F1-Measure (%)
CNN	45.75	44.50	45.00
1D Self-Attention CNN	86.66	78.00	78.50

**Table 7:** Comparison of accuracy of different models and training strategies.

Basic Model	Unworn Accuracy (%)	Worn Accuracy (%)
CNN	76	19
1D Self-Attention CNN	100	56
CNN + incremental learning	/	45.75
1D Self-Attention CNN + incremental learning	/	86.66

**Table 8:** Accuracy comparison between standard 1D Self-Attention CNN and its incremental learning variant under different rotational speeds.

Rotational Speed (rpm)	1D Self-Attention CNN Accuracy (%)	1D Self-Attention CNN + Incremental Learning Accuracy (%)
300	98.67	84.76
600	100.00	86.66
900	100.00	94.29

## Data Availability

The data presented in this study are available on request from the corresponding author due to privacy/ethical restrictions.
